# A Dynamic Self-Adjusting System for Permanent Magnet Synchronous Motors Using an Improved Super-Twisting Sliding Mode Observer

**DOI:** 10.3390/s25123623

**Published:** 2025-06-09

**Authors:** Yanguo Huang, Yingmin Xie, Weilong Han

**Affiliations:** 1 School of Electrical Engineering and Automation, Jiangxi University of Science and Technology, Ganzhou 341000, China; 6120230625@mail.jxust.edu.cn (Y.X.); 6720230661@mail.jxust.edu.cn (W.H.); 2 Jiangxi Provincial Key Laboratory of Multidimensional Intelligent Perception and Control, Jiangxi University of Science and Technology, Ganzhou 341000, China

**Keywords:** permanent magnet synchronous motor, sensorless control, sliding mode observer, error factor, neural network

## Abstract

To enhance the robustness of sensorless control in permanent magnet synchronous motors (PMSMs) under parameter mismatches, this paper proposes a novel sliding mode observer (SMO) that automatically adjusts the error factor. The purpose is to enable the precise observation of rotor position in PMSMs while simultaneously suppressing chattering and simplifying the design process. First, an SMO based on an adjustable error factor is designed, which reduces chattering and eliminates the need for a low-pass filter (LPF). The impact of the error factor within the SMO is then analyzed, including its effects on the estimation of current, speed, and position, and a method for determining the error factor based on these estimated values is introduced. This method uses a neural network algorithm to balance chattering suppression with high control accuracy. Finally, a neural network-based self-adjusting SMO model is proposed to automatically adjust the error factor based on motor operating conditions. Simulation and experimental results demonstrate the feasibility and effectiveness of this approach.

## 1. Introduction

The permanent magnet synchronous motor (PMSM) has numerous advantages, such as compact size, simple structure, and high efficiency [1], making it widely used in fields such as medical equipment and aerospace [2,3]. In these applications, the control accuracy and response speed of PMSMs directly impact the performance and reliability of the system. Various nonlinear control methods, such as adaptive control [4], model predictive control [5], and sliding mode control (SMC) [6], have been extensively applied in PMSM drivers. Research efforts primarily focus on developing advanced algorithms that adapt to the nonlinear behavior of PMSMs [7]. Achieving high-precision speed and position control has become a key area of study.

Traditional PMSM control methods typically rely on position sensors to obtain rotor position information in real-time [8], thereby enabling precise motor control. Examples include encoders and Hall sensors [9]; however, position sensors increase system complexity and cost, and their reliability is often compromised in harsh environments characterized by high temperatures, vibrations, and dust, which severely affect motor control accuracy. Consequently, the development of sensorless control technologies has become a hot research topic. Sliding mode control (SMC) is a powerful nonlinear control technique that has been widely used for speed and position control in PMSM systems [10], especially in sensorless control applications [11]. The sliding mode observer (SMO) [12], as an implementation of sliding mode control, can estimate rotor speed and position information by estimating back electromotive force (EMF) or magnetic flux, offering fast dynamic response, insensitivity to parameter variations, and strong robustness [13,14]. However, early SMO methods employed the sign function as a switching function [15]. Although this approach is simple, it can lead to significant chattering phenomena, adversely affecting the stability and precision of the control system.

To address the chattering issue, researchers have proposed various improvement strategies [16]. For example, the introduction of an LPF can smooth the output signal; however, using an LPF in signal demodulation increases the order of the position estimation system and adds unstable zero points, leading to poor system stability [17]. To mitigate this, the literature [18] details the introduction of a negative coefficient LPF in parallel with the traditional CCF (TR-CCF) loop, complicating the system further. Although these methods have improved system performance to some extent, they have also made the system more complex. Therefore, researchers have shifted their focus to improving the design of sliding mode gains and switching functions.

Regarding improved sliding mode gain, the literature [19] details the design of a variable sliding mode gain related to speed, which attempts to reduce chattering and enhance system robustness. The literature [20] describes the use of an independent sliding mode gain with a correction term, but its effectiveness under high-frequency conditions was inadequate. The literature [21] details how the sigmoid function was used instead of the sign function, but the smoothness of the sigmoid function may lead to a decrease in control accuracy as the system approaches the target state. The literature [22] describes the employment of a piecewise linear function to replace the sign function, but the use of two-phase filtering and phase compensation made the system more complex. The literature [23] proposed using a piecewise power function to replace the traditional sign function to eliminate chattering; however, this method still has certain limitations. The literature [24] and the literature [8], respectively, detail the adoption of a novel hyperbolic function and a hyperbolic tangent switching function with self-adjusting shape coefficients, which can reduce chattering phenomena in SMOs without needing to reintroduce LPF to mitigate the adverse effects of chattering on position estimation accuracy. The literature [25] details the use of an exponential input function and the employment of fuzzy control to obtain the boundary layer method, but the system also exhibited randomness. The literature [26] details the introduction of a variable boundary layer sine saturation function and proposed a method to determine the boundary layer based on the rate of change in estimated current error; however, selecting the boundary layer still requires considerable effort.

Although existing methods have achieved some success in chattering suppression, there remains a lack of effective guidelines for selecting the boundary layer, often relying on multiple simulations or experiments to determine it. This not only increases design complexity but also restricts engineering applications. The literature [25,26] provided some regularity analysis for boundary layer selection but did not further establish algorithm models to summarize the patterns. The literature [27] details the design of a gradual angle-based saturation function for modified composite sliding mode control (MCSMC) to achieve dynamic changes in the boundary layer, but the introduced state trajectory’s gradual angle range is quite small, which may limit its applicability. The literature [28] describes the employment of a multilayer perceptron (MLP)-based neural network to directly generate the switching term of the sliding mode control law, replacing the traditional sign function. Theoretically, this approach can suppress chattering through the nonlinear mapping capability of the network. However, this method requires parallel integration of two independent networks in the control loop (processing *d*-axis and *q*-axis current errors separately) and introduces additional phase synchronization filters, leading to increased structural complexity in the system. Moreover, the compatibility between neural network weight updates and sliding mode stability has not been rigorously proven. To address these issues, the subsequent literature [29] proposes an improved scheme utilizing a radial basis function (RBF) neural network with adaptive laws for online adjustment of network center parameters, aiming to reduce reliance on offline training. Nonetheless, this method still exhibits notable limitations: the width of the RBF network’s basis functions requires empirical presetting, which may lead to increased approximation errors under motor parameter perturbations. No saturation constraint mechanism is designed for the network output, potentially causing current oscillations during abrupt load changes due to output exceeding permissible limits.

To suppress chattering in SMOs while ensuring control precision and achieving the goal of automatic adjustment to reduce design workload, this paper presents the following innovative contributions:

1. An improved error function replaces the piecewise function, enabling automatic adjustment of the system through the adjustment of the error factor without considering the selection of the boundary layer, significantly reducing the workload.
2. A comprehensive analysis of the impact of the error factor in SMOs is conducted, including observations of estimated current, speed, and position, summarizing corresponding patterns. Specifically, the chattering in SMOs decreases and then increases with the increase in the error factor, while the estimation error also decreases and then increases with the error factor. As the SMO state moves away from the sliding mode surface, the accuracy of the estimated position declines, ultimately reducing control precision. Based on this pattern, selection criteria for the error factor are derived, emphasizing the need to balance chattering suppression and control precision maintenance.
3. Combining the patterns and selection criteria from the error factor impact analysis, a neural-network-error-factor self-adjusting SMO model is designed. This method not only considers the balance between chattering and control precision but also significantly reduces design workload, allowing for self-adjustment of the error factor based on actual working conditions. Finally, simulations and experimental validations in the MATLAB/Simulink environment demonstrate that the proposed method exhibits good feasibility and effectiveness, providing new ideas and methods for sensorless control technologies in PMSMs. This research not only enriches the theoretical framework of sliding mode observers but also offers strong support for practical engineering applications.

## 2. Traditional Sliding Mode Observation Method

### 2.1. Traditional Sliding Mode Observer

**Figure 1:**
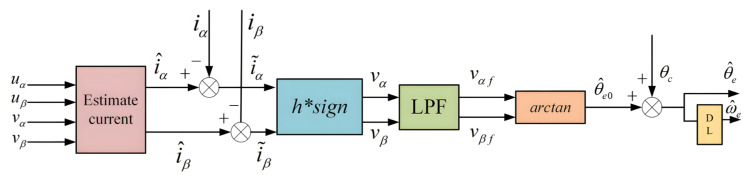
Schematic diagram of the conventional SMO.

In modern electric drive systems, PMSMs have garnered significant attention due to their high efficiency and excellent control characteristics. To further enhance their control accuracy and robustness, SMOs have been widely used in the control systems of PMSMs as an effective state estimation method. The structure of the traditional Super-Twisting SMO is shown in Figure 1.

The motor is assumed to be a surface-mounted PMSM (SPMSM) with a nonsalient-pole rotor, satisfying $l_{d}=l_{q}$. The mathematical model of the motor in the *α* − *β* axis coordinate system can be expressed as:

(1) $$\left[\begin{array}{c} u_{\alpha }\\ u_{\beta } \end{array}\right]=\left[\begin{array}{cc} r+Pl_{d} & \omega_{e}\left(l_{d}-l_{q}\right)\\ -\omega_{e}\left(l_{d}-l_{q}\right) & r+Pl_{d} \end{array}\right]\left[\begin{array}{c} i_{\alpha }\\ i_{\beta } \end{array}\right]+\left[\begin{array}{c} E_{\alpha }\\ E_{\beta } \end{array}\right]$$

In the expression, $u_{a}$ and $u_{\beta }$ are the voltage components in the α−β axis coordinate system; $i_{\alpha }$ and $i_{\beta }$ are the current components in the α−β axis coordinate system; and $E_{\alpha }$ and $E_{\beta }$ are the EMF components of the motor, which include the rotor position information. The expression is given by:

(2) $$\left[\begin{array}{c} E_{\alpha }\\ E_{\beta } \end{array}\right]=\left[\begin{array}{c} (l_{d}-l_{q})(\omega_{e}i_{d}-Pi_{q})+\omega_{e}\lambda_{f} \end{array}\right]\left[\begin{array}{c} -\sin\theta_{e}\\ \cos\theta_{e} \end{array}\right]$$

The current error in the *α* − *β* axes is defined as the sliding mode surface:

(3) $$M=\left(\begin{array}{c} s_{\alpha }\\ s_{\beta } \end{array}\right)=\left(\begin{array}{c} {\hat{\iota }}_{\alpha }-i_{\alpha }\\ {\hat{\iota }}_{\beta }-i_{\beta } \end{array}\right)$$

Rewrite Equation (3) as the current equation:

(4) $$P\left[\begin{array}{c} i_{\alpha }\\ i_{\beta } \end{array}\right]=\frac{1}{l_{d}}\left(N\left[\begin{array}{c} i_{\alpha }\\ i_{\beta } \end{array}\right]+\left[\begin{array}{c} u_{\alpha }\\ u_{\beta } \end{array}\right]-\left[\begin{array}{c} E_{\alpha }\\ E_{\beta } \end{array}\right]\right)$$

The expression for the coefficient matrix N is:

(5) $$N=\left[\begin{array}{cc} -r_{s} & -\omega_{e}(l_{d}-l_{q})\\ \omega_{e}(l_{d}-l_{q}) & -r_{s} \end{array}\right]$$

To obtain $E_{\alpha }$ and $E_{\beta }$, the construction of SMO is as follows:

(6) $$p\left[\begin{array}{c} {\hat{\iota }}_{\alpha }\\ {\hat{\iota }}_{\beta } \end{array}\right]=\frac{1}{l_{d}}\left(N\left[\begin{array}{c} {\hat{\iota }}_{\alpha }\\ {\hat{\iota }}_{\beta } \end{array}\right]+\left[\begin{array}{c} u_{\alpha }\\ u_{\beta } \end{array}\right]-\left[\begin{array}{c} v_{\alpha }\\ v_{\beta } \end{array}\right]\right)$$

In the equation, ${\hat{i}}_{\alpha }$ and ${\hat{i}}_{\beta }$ are the current components observed by the SMO, where the superscript “∧” indicates estimated signal and $v_{\alpha }$ and $v_{\beta }$ are the control inputs of the SMO, which can be expressed by Equation (7):

(7) $$\left[\begin{array}{c} v_{\alpha }\\ v_{\beta } \end{array}\right]=\left[\begin{array}{c} k\mathrm{sign}({\hat{\iota }}_{\alpha }-i_{\alpha })\\ k\mathrm{sign}({\hat{\iota }}_{\beta }-i_{\beta }) \end{array}\right]$$

In the equation, “*sign*” represents the sign function and “*k*” denotes the sliding mode gain. Let ${\tilde{\iota }}_{\alpha }$ = ${\hat{\iota }}_{\alpha }-i_{\alpha }$ and ${\tilde{\iota }}_{\beta }$ = ${\hat{\iota }}_{\beta }-i_{\beta }$ represent the current estimation errors, where the tilde symbol “∼” indicates the observation error. Subtracting Equation (4) from Equation (6) yields:

(8) $$P\left[\begin{array}{c} {\tilde{\iota }}_{\alpha }\\ {\tilde{\iota }}_{\beta } \end{array}\right]=\frac{1}{l_{d}}\left(N\left[\begin{array}{c} {\tilde{\iota }}_{\alpha }\\ {\tilde{\iota }}_{\beta } \end{array}\right]+\left[\begin{array}{c} E_{\alpha }-\nu_{\alpha }\\ E_{\beta }-\nu_{\beta } \end{array}\right]\right)$$

The sliding mode gain *k* in Equation (7) determines whether the estimated current components in Equation (6) can converge to the actual current components $i_{\alpha }$ and $i_{\beta }$, as well as the convergence speed. To ensure the stability of the SMO, *k* should satisfy the following conditions:

(9) $$k>\max\{|E_{\alpha }|,|E_{\beta }|\}$$

To suppress chattering ${\hat{e}}_{\alpha }$ and ${\hat{e}}_{\beta }$ are filtered using an LPF to remove high-frequency harmonics before being used for position calculation. According to Equation (2), the back EMF in the α−β axis contains cosine and sine information related to the position. Therefore, the estimated position information can be obtained using the arctangent function, and the estimated speed can be further derived through differentiation. The expression for the estimated position is shown in Equation (10):

(10) $${\hat{\theta }}_{eo}=\arctan\left(-\frac{\nu_{\alpha }}{\nu_{\beta }}\right)$$

Due to the introduction of the LPF, phase lag will occur, so position compensation needs to be implemented based on the speed and the bandwidth of the LPF. The compensation angle $\theta_{c}$ can be calculated using the following formula:

(11) $$\theta_{c}=\arctan\left(\frac{{\hat{\omega }}_{e}}{\omega_{c}}\right)$$

In the equation, ${\hat{\omega }}_{e}$ represents the estimated speed, and $\omega_{c}$ is the bandwidth of the LPF. The compensated estimated position and estimated speed of the rotor can be expressed as follows:

(12) $${\hat{\theta }}_{e}={\hat{\theta }}_{eo}+\theta_{c},{\hat{\omega }}_{e}=\frac{d{\hat{\theta }}_{e}}{dt}$$

### 2.2. Sliding Mode Observer

Fundamentally, the lag problem caused by the LPF in traditional SMOs mainly arises from the chattering handling method. Therefore, eliminating the use of low-pass filters is key to solving this issue. For boundary-layer-variable sliding mode observers, although low-pass filters and additional angle compensation are no longer needed, a significant amount of computation is still required to select appropriate sliding surfaces and boundary layer widths. This may lead to a reduction in the observer’s response speed or exacerbate chattering phenomena. To address this, this paper proposes a novel sliding mode observer based on an error function, with its expression given in Equation (13). Compared to traditional saturation functions, this error function has better continuity within the boundary layer, and its convergence speed is faster than that of other commonly used switching functions. The improved error function F() can be expressed as:

(13) $$F(s)=F(zx)=\mathrm{erf}(zx)=\frac{2}{\pi }\int_{0}^{s}e^{-t^{2}}dt$$

The selection of the sliding mode surface is the same as that of traditional sliding mode observers, satisfying Equation (3). Therefore, the control input of the sliding mode observer can be rewritten as:

(14) $$\left[\begin{array}{c} v_{\alpha }\\ v_{\beta } \end{array}\right]=\left[\begin{array}{c} k\cdot F(s_{\alpha })\\ k\cdot F(s_{\beta }) \end{array}\right]$$

When the system reaches the sliding mode surface, it satisfies:

(15) $$\dot{s}=s=0$$

Based on the EMF expressions for the α−β axes and the control input expression of the SMO, combined with the conditions for the SMO to reach the sliding mode surface, it can be concluded that when the SMO converges, the observation error of the EMF approaches 0, satisfying Equation (16):

(16) $$\left\{\begin{array}{l} E_{\alpha }-\nu_{\alpha }\approx 0\\ E_{\beta }-\nu_{\beta }\approx 0 \end{array}\right.$$

Although the aforementioned method has eliminated the use of the LPF while ensuring the convergence speed of the system and has reduced the chattering of the SMO, the impact of 𝑍 in the SMO has not been analyzed, and there is still room for improvement in the selection method of 𝑍 in F(s).

### 2.3. Stability Analysis

Stability analysis is primarily used to evaluate the behavior of dynamic systems or control systems under various conditions, ensuring that the system remains stable when subjected to disturbances or changes in initial conditions, thereby avoiding undesirable oscillations or loss of control. In order to ensure the stability of the designed observer, it is necessary to base it on the following Lyapunov function:

(17) $$V=\frac{1}{2}\cdot s^{T}\cdot s=\frac{1}{2}{\tilde{\iota }}_{\alpha }^{2}+\frac{1}{2}{\tilde{\iota }}_{\beta }^{2}$$

The equality of *d*-axis and *q*-axis inductances indicates a nonsalient-pole PMSM. In such motors, the rotor magnetic circuit is symmetric, resulting in negligible reluctance torque, and the electromagnetic torque is predominantly governed by the permanent magnet excitation torque. Clearly, *V* > 0. According to the Lyapunov stability criterion, we can conclude that the SMO can reach a stable state only if we derive *dV*/*dt* < 0. Taking the time derivative of Equation (17) gives:

(18) $$\frac{dV}{dt}=s^{T}\frac{dS}{dt}={\tilde{\iota }}_{\alpha }\frac{d{\tilde{\iota }}_{\alpha }}{dt}+{\tilde{\iota }}_{\beta }\frac{d{\tilde{\iota }}_{\beta }}{dt}$$

Substituting Equation (8) and Equation (14) yields:

(19) $$\frac{dV}{dt}=[{\tilde{\iota }}_{\alpha }\;\;\;\;{\tilde{\iota }}_{\beta }]\frac{1}{L_{d}}\left(N\left[\begin{array}{c} {\tilde{\iota }}_{\alpha }\\ {\tilde{\iota }}_{\beta } \end{array}\right]+\left[\begin{array}{c} E_{\alpha }-kF({\tilde{\iota }}_{\alpha })\\ E_{\beta }-kF({\tilde{\iota }}_{\beta }) \end{array}\right]\right)$$

Expanding Equation (19) gives:

(20) $$\frac{dV}{dt}=\frac{A}{L_{d}}\left({\tilde{\iota }}_{\alpha }^{2}+{\tilde{\iota }}_{\beta }^{2}\right)+\frac{1}{L_{d}}\left[(E_{\alpha }-kF({\tilde{\iota }}_{\alpha })){\tilde{\iota }}_{\alpha }+\left(E_{\beta }-kF({\tilde{\iota }}_{\beta })\right){\tilde{\iota }}_{\beta }\right]$$

To maintain the stability of the SMO, both terms must be less than 0. Based on the coefficient matrix *N*, the first term is less than 0. In order for the second term to be less than 0, the observer gain must satisfy the following inequality condition:

(21) $$k>\max\left(\left|\frac{E_{\alpha }}{F({\tilde{\iota }}_{\alpha })}\right|,\left|\frac{E_{\beta }}{F({\tilde{\iota }}_{\beta })}\right|\right)$$

when s=0, the system strictly reaches the sliding manifold (${\tilde{i}}_{\alpha }=0,{\tilde{i}}_{\beta }=0$), and the observed back-electromotive force (BEMF) errors satisfy $E_{\alpha }-v_{\alpha }\approx 0,\;E_{\beta }-v_{\beta }\approx 0$.

At this state, the BEMF errors converge to zero: $E_{\alpha }\approx v_{\alpha }=kF\left(s_{\alpha }\right),\;E_{\beta }\approx v_{\beta }=kF\left(s_{\beta }\right)$. Since $F\left(s\right)=0$, it follows that $E_{\alpha }\approx 0,\;E_{\beta }\approx 0$. From inequality (21), when $F\left(s\right)=0$, both the numerator ($E_{\alpha },\;E_{\beta }$) and denominator ($F(i_{\alpha })$, $F(i_{\beta })$) approach zero (due to system convergence), resulting in a **0/0** indeterminate form.

The continuity of F(⋅) (e.g., $\mathrm{erf}(s)\approx \frac{2}{\sqrt{\pi }}s$ for s→0) ensures the ratio $\frac{E}{F(\tilde{i})}$ does not diverge but converges to a finite value.

## 3. Selection of the Error Factor

### 3.1. Impact Analysis of the Error Factor

**Figure 2:**
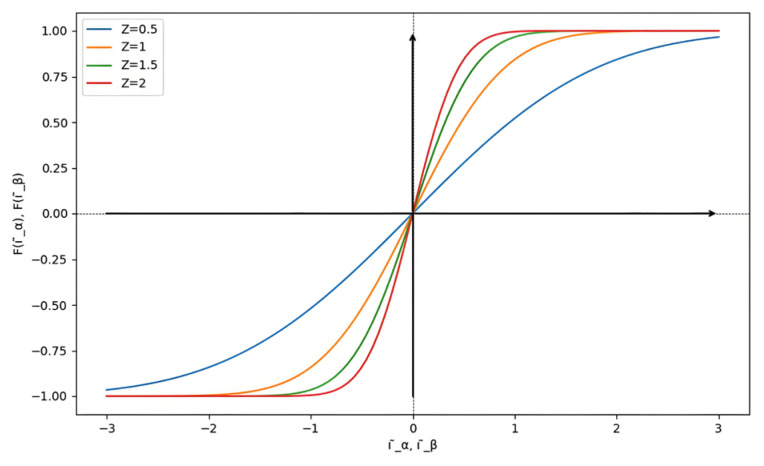
The curve of the error function corresponds to different *Z* values.

In the design of the SMO, the choice of the sign function is particularly important as it is closely related to the suppression of chattering and the control precision of the SMO. This paper employs an improved error function to analyze the impact of varying the value of *Z* on the entire system. This includes the observed EMF, estimated current, estimated speed, and estimated position. An analysis of the selection of *Z* under different conditions is conducted, with the corresponding sign functions for different *Z* values illustrated in Figure 2.

### 3.2. Establishing the Error Factor Using Neural Network Algorithms

**Figure 3:**
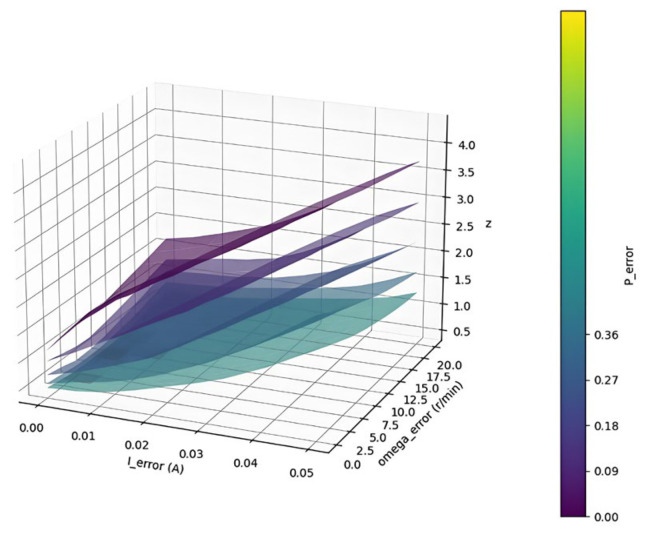
Shows the optimal values of *Z* under different variables.

To establish a model for the error factor, the relationship between different speeds, currents, position errors, and the magnitude of the error factor is analyzed. In practical scenarios, the value of *Z* can be adjusted in real-time based on varying speeds and other information, thereby optimizing the estimation error and chattering conditions of the system. A model concerning the value of *Z* is constructed within the SMO to minimize the error between the predicted values and the actual speed, current, and position. Figure 3 illustrates the optimal *Z* values across various variables.

Based on the analysis of experimental results, once the errors in speed, current, and position are determined, there exists an optimal error factor that minimizes the overall error. When paired with a neural network model, the PMSM system can effectively reduce errors by adjusting the error factor in real time during actual operating conditions.

## 4. Simulation Verification

**Figure 4:**
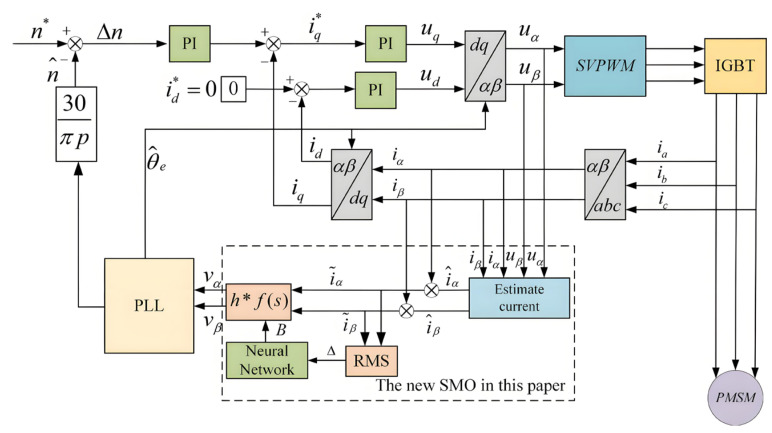
PMSM sliding mode observer sensorless control system.

To validate the feasibility of the proposed error function as a compliance function and to assess the effectiveness of the error factor adjustment in suppressing chattering and enhancing model accuracy under varying operating conditions, this section analyzes the system’s performance under changing speed and sudden load increments. The PMSM sliding mode observer sensorless model constructed using MATLAB R2022a/Simulink is illustrated in Figure 4.

A vector control method is employed with a sampling period of 0.1 s, and the relevant simulation parameters are listed in Table 1. The stator winding resistance ($R_{s}$) represents the per-phase value measured at 25 °C using a four-terminal method, with a nominal value of 2.875 Ω/per phase.

**Figure 5:**
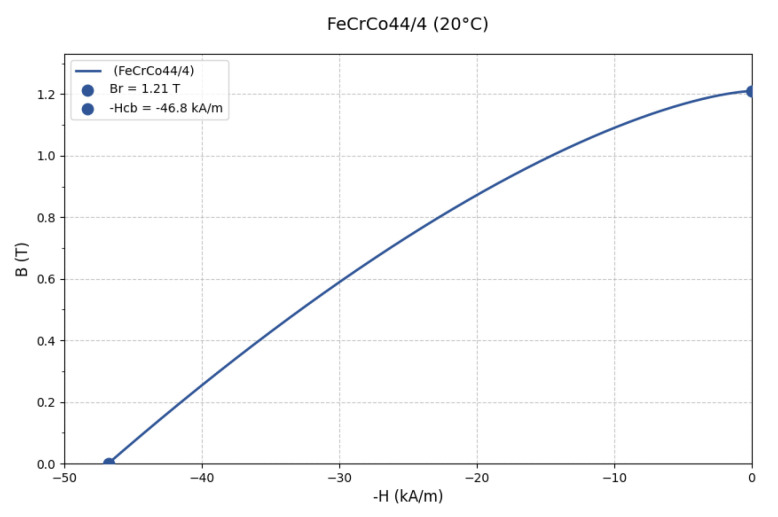
The demagnetization curve of FeCrCo44/4.

Figure 5 shows the hysteresis loop based on FeCrCo44/4 permanent magnetic material at 20 °C.

The diagram shows its coercivity (Hc = 46.8 kA/m) and residual flux density (Br = 1.21 T). This is an iron-chromium-cobalt alloy that has significant advantages in permanent magnet applications due to its high coercivity and good temperature stability.

### 4.1. Speed Variation Analysis

**Figure 6:**
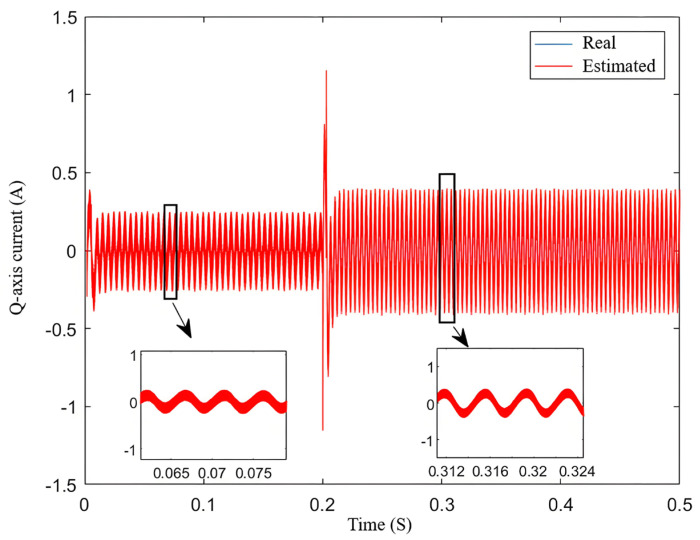
Variation in *q*-axis current.

In the no-load condition, the reference speed is set to increase from 800 r/min to 1000 r/min at 0.2 s and then return to 800 r/min at 0.5 s. The simulation results are shown in Figure 6.

Figure 6 shows the variation in the *q*-*axis* current. The blue line is the real speed, and the red line is the estimated speed. It can be seen from the Figure that the estimated current closely follows the actual current, and the error increases as the speed rises.

**Figure 7:**
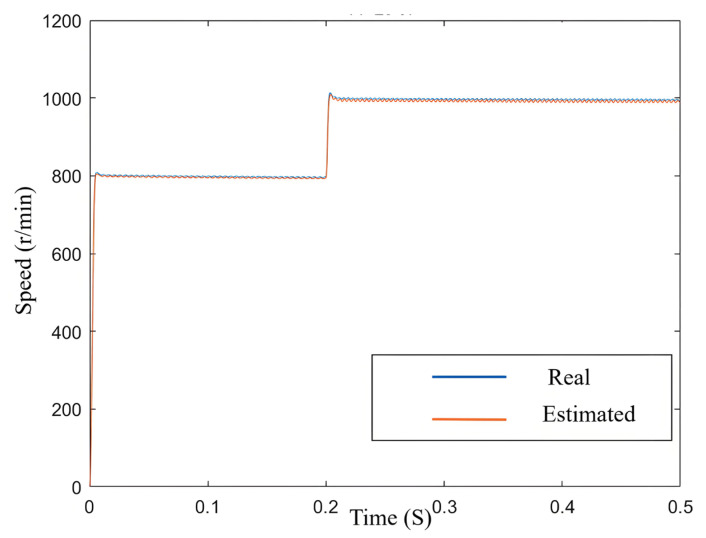
Variation in estimated speed and actual speed.

As observed in Figure 7, the estimated speed closely aligns with the actual speed, and the chattering of the estimated speed is minimal.

**Figure 8:**
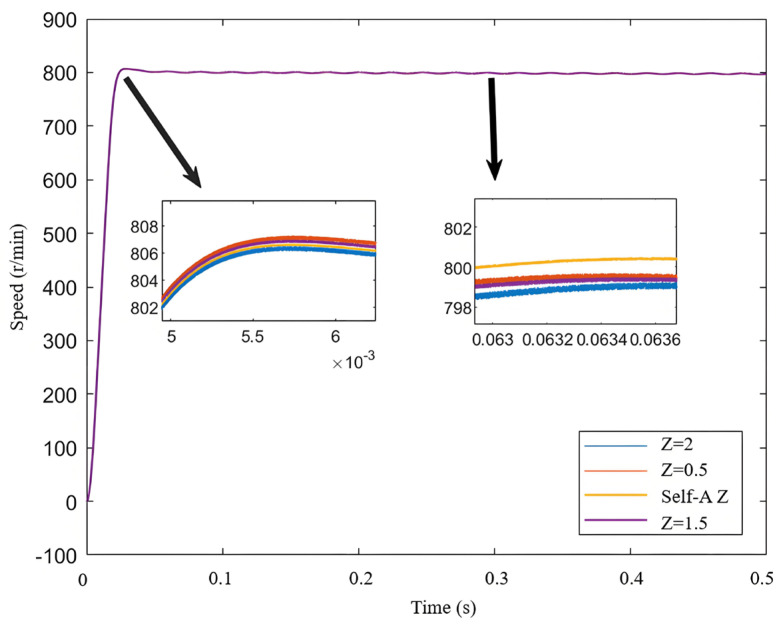
Estimated speed curves for different *Z* values.

Figure 8 illustrates the estimated speed over time under different error factors based on the neural network. The initial speed is set to 800 r/min.

As shown by the yellow line in the figure, although the neural network self-regulating error factor does not provide the fastest response speed, its exhibited characteristics are noteworthy. This method achieves a good balance between oscillation suppression and control accuracy, indicating its adaptability and stability under dynamic load conditions. In the fixed error factor SMO, since the error factor is constant, the system struggles to adjust flexibly when faced with load changes, resulting in larger speed errors and more pronounced oscillations. In contrast, the neural network self-regulating SMO continuously learns and adapts, allowing it to dynamically adjust its control strategy, thereby maintaining smaller speed errors during load variations.

**Figure 9:**
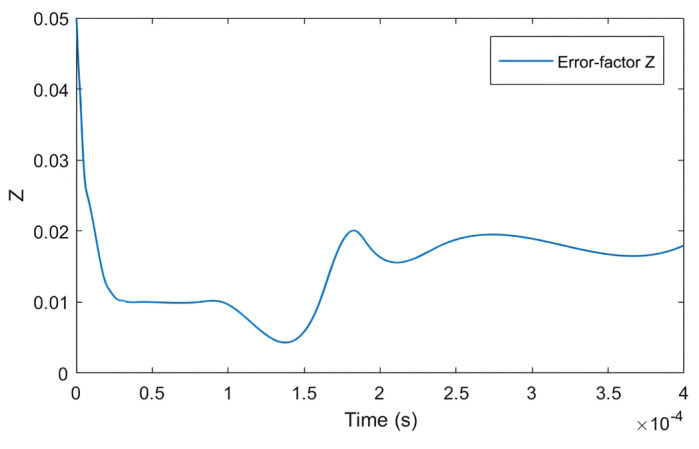
Variation in *Z* value over time during speed changes.

From Figure 9, it can be seen that the neural network self-adjusting *Z* value model can calculate the optimal *Z* value based on real-time speed errors and continuously update it. This allows the PMSM system to achieve optimal estimated speed during speed observation. Thus, it can be concluded that the neural-network-error-factor self-adjusting SMO designed in this paper is feasible during speed variations.

### 4.2. Sudden Increase Load Analysis

#### 4.2.1. Simulation Verification Under Sudden Load

**Figure 10:**
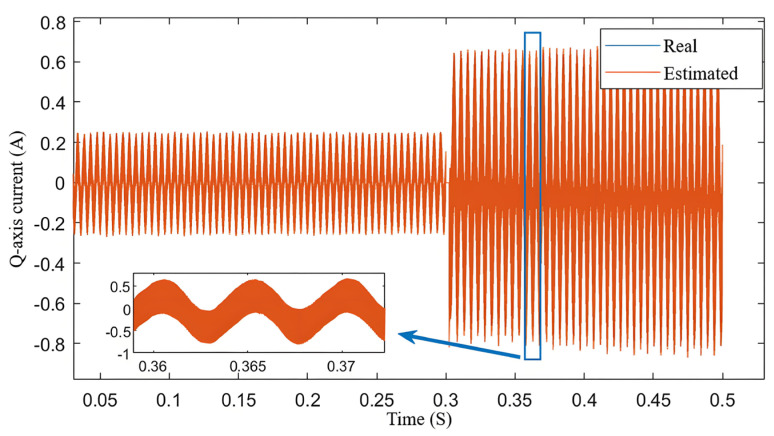
Variation in estimated current and estimated current error during sudden.

Given that $l_{d}=l_{q}$, the maximum torque per ampere (MTPA) control strategy degenerates to maintaining id = 0, which significantly simplifies the implementation The speed is set to 800 r/min, with a no-load condition for the first 0.3 s, followed by a sudden load increase of 5 N·m at 0.3 s, as shown in Figure 10. 

It can be clearly seen from Figure 10 that although the system faces significant disturbances at the moment of a sudden load increase, the estimated current can effectively follow the changes in the actual current. The blue box in Figure 10 is a *q*-axis current from 0.36 s to 0.37 s. The system responds quickly to load variations, and furthermore, the fluctuation amplitude of the estimated current is very small. This further demonstrates the stability and reliability of the method in dynamic environments.

**Figure 11:**
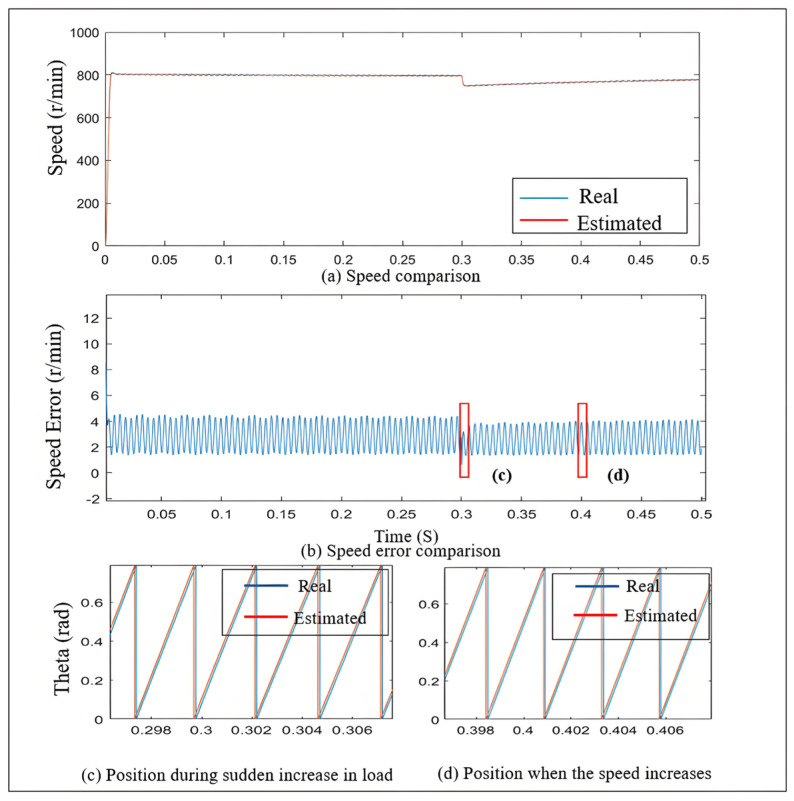
Speed curve and estimated position error curve during sudden load increase.

Figure 11 shows that at the moment of sudden load increase, the difference between the estimated speed and the actual speed is very small, and the oscillation phenomenon is also relatively mild. Under steady-state conditions, the estimated position error remains below 0.02 rad. These results fully validate the effectiveness and reliability of the SMO proposed in this paper under sudden load conditions.

**Figure 12:**
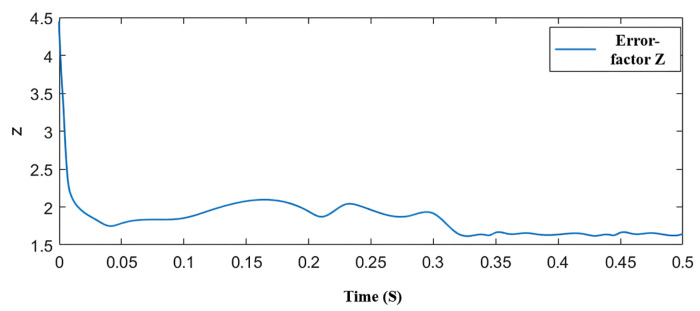
Variation in Z during sudden load increase.

Figure 12 shows the variation in *Z* during a sudden load increase. From the figure, it can be analyzed that under no-load conditions at a speed of 800 r/min, the error factor *Z* is approximately 1.75. After the sudden load increase at 0.3 s, according to the self-regulating algorithm, the *Z* value decreases to 1.62. When the load increases, the estimated current error decreases, and under the original boundary layer, the output *F*(*s*) decreases. The red boxes in the figure are the real position and the estimated position at 0.3 s and 0.4 s, respectively.

Since the speed remains unchanged, the EMF and the sliding mode gain k also remain constant, thus, the corresponding observed EMF *να* does not change.

To achieve this, it is necessary to reduce the *Z* value to increase the slope, thereby keeping the output of *F*(*s*) constant and ensuring that the observed EMF *να* remains unchanged under sudden load increases. By lowering the *Z* value, the system can respond more quickly to load changes, reducing the instantaneous fluctuations caused by sudden load increases and effectively suppressing oscillation phenomena. This adjustment also enhances the system’s robustness against external disturbances, ensuring that good dynamic performance and control effects are maintained under various operating conditions.

#### 4.2.2. Effectiveness Verification During Sudden Load Increase

**Figure 13:**
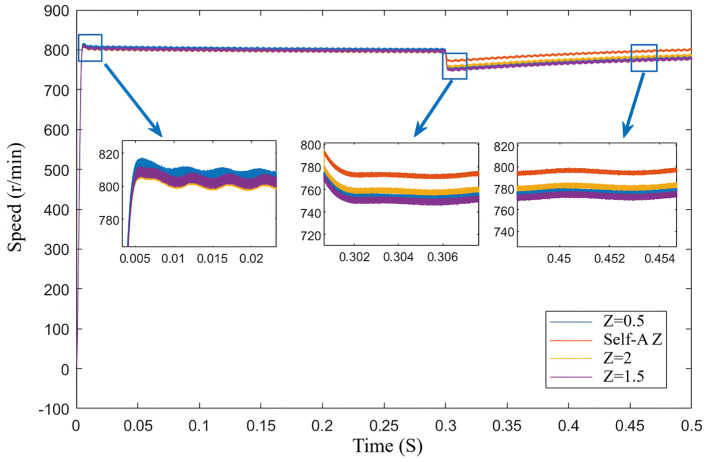
Speed curves during sudden load increase for different *Z* values.

**Figure 14:**
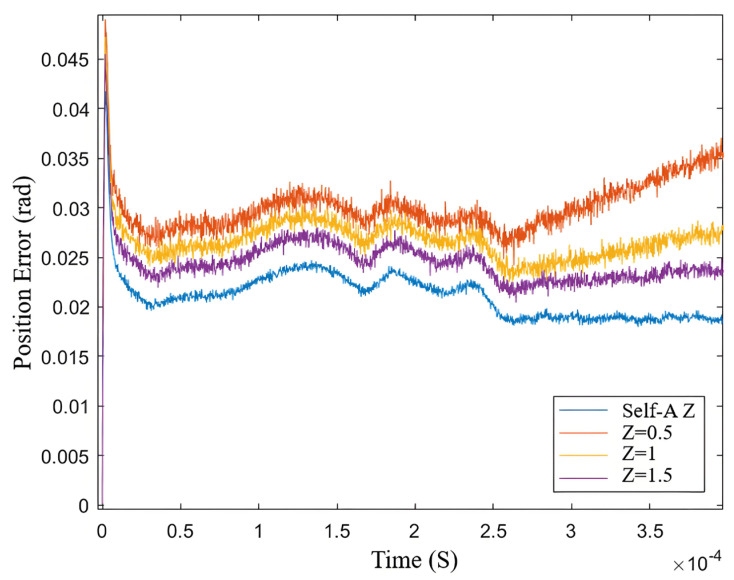
Estimated position error curves during sudden load increase for different *Z* values.

To verify its effectiveness, a comparison is made between the self-adjusting *Z* value and the fixed *Z* value regarding the observation effects during a sudden load increase of 5 N·m. The simulation results are shown in Figure 13 and Figure 14.

From the speed curves in Figure 13, The blue box is the rotation speed at different z values. it can be observed that when the *Z* value is too high, overshoot occurs; although dynamic performance is improved, steady-state chattering is significant. Conversely, when the *Z* value is too low, load resistance is compromised. As shown in Figure 14, the estimated position error under the neural network self-adjusting *Z* value is consistently lower than that under the fixed *Z* value. As indicated by the orange line, when *Z* is too low, the system’s load-bearing capacity is affected, leading to divergence after the sudden load increase. This result demonstrates the effectiveness of the improved SMO and the neural network self-adjusting *Z* value method proposed in this paper.

As shown in Figure 14, the SMO with a neural network-based self-adjusting *Z* value demonstrates significantly better performance in estimating position errors compared to the fixed *Z* value SMO. With the dynamic adjustment of the *Z* value, the estimated position error consistently remains at a low level, indicating higher accuracy and stability.

The result suggests that the neural network self-adjustment mechanism can effectively adapt to changes in system states, thereby optimizing the observer’s performance.

As indicated by the orange line, when the *Z* value is set too low, the system’s load-bearing capacity is significantly affected under sudden load changes, leading to divergence. This phenomenon underscores the importance of selecting an appropriate *Z* value; while a smaller *Z* value may enhance response speed in certain situations, it can also compromise the system’s robustness. This finding validates the effectiveness of the proposed method that combines the improved sliding mode observer with a neural network for self-adjusting the *Z* value.

## 5. Experimental Verification

**Figure 15:**
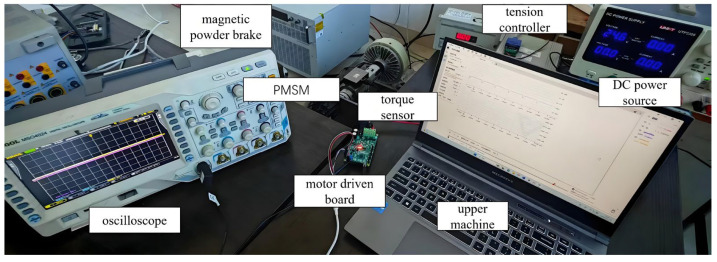
Experimental platform.

The main control chip in the motor control board is the STM32G431CBU6, STM32G431CBU6 equipment manufacturer is STMicroelectronics, headquartered in Chloe, France, and the inverter circuit employs the FD6288T module, FD6288T from Fortior Tech, Shenzhen, China, with a DC bus voltage supply of 24 V. The motor test platform is shown in Figure 15.

### 5.1. Verification of Speed Variation for Self-Adjusting Error Factor SMO

#### 5.1.1. Comparison of Speed Increase and Decrease Experiments

**Figure 16:**
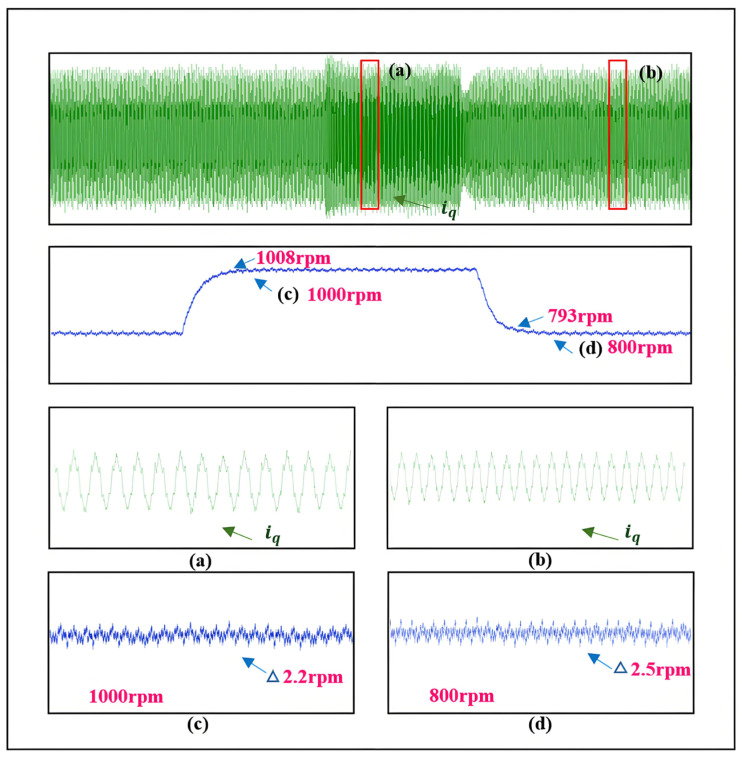
Experimental results during speed increase and decrease. (**a**) is the *q*-axis current change during acceleration. (**b**) is the *q*-axis current change during deceleration. (**c**) is the speed change during acceleration. (**d**) is the speed change during deceleration.

To verify the effectiveness of the selected *Z* value, this section compares the observation effects of the neural network self-adjusting *Z* value and the fixed *Z* value under the SMO at a speed of 800 r/min. The comparative experimental results are shown in Figure 16.

The rotational speed accurately tracks the reference input throughout the operation, with overshoot 1008 rpm peak value and response time matching simulation predictions, achieving rapid stabilization, as quantitatively illustrated in the figure. Meanwhile, the estimated speed demonstrates reliable convergence to the actual rotor position, maintaining speed tracking errors within 2.2–2.5 rpm during both acceleration and deceleration transients.

#### 5.1.2. Verification of Effectiveness of Self-Adjusting *Z* Value During Speed Increase

**Figure 17:**
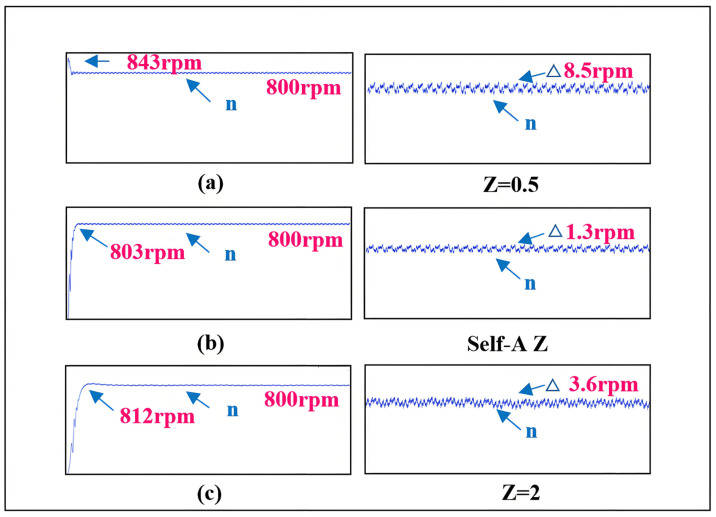
Comparison of speed for different *Z* values. (**a**) The rotational speed and rotational speed error when *Z* = 0.5. (**b**) The rotational speed and rotational speed error when Self-A *Z*. (**c**) The rotational speed and rotational speed error when *Z* = 2.

The comparison of estimated speed aligns with the simulation results. In terms of dynamic performance, as *Z* increases, the rise time of the speed increases while the overshoot gradually decreases. Regarding steady-state performance, as *Z* increases, the steady-state speed fluctuations gradually diminish. Compared to the neural network self-adjusting error factor, the steady-state-error mean values under both non-optimal boundary layers increase. This is illustrated in Figure 17.

Combining Figure 17 with the error function curves corresponding to different *Z* values in Figure 2, it can be analyzed that the comparison of estimated speeds aligns with the simulation results. In terms of dynamic performance, as *Z* increases, the rise time of the speed increases while the overshoot gradually decreases. Regarding steady-state performance, as *Z* increases, the steady-state speed fluctuations gradually diminish. At 800 r/min, the comparison of estimated position and estimated position error also aligns with the previous analysis and simulation, showing that as *Z* increases, the fluctuations in the estimated position under steady-state operation significantly decrease. Compared to the neural network self-adjusting error factor, the steady-state-error mean values under both non-optimal boundary layers increase. The experimental results in Figure 17 demonstrate the effectiveness of the neural-network-error-factor self-adjusting method proposed in this paper in suppressing chattering and enhancing accuracy.

#### 5.1.3. Verification of Effectiveness of the Novel SMO During Speed Increase and Decrease

**Figure 18:**
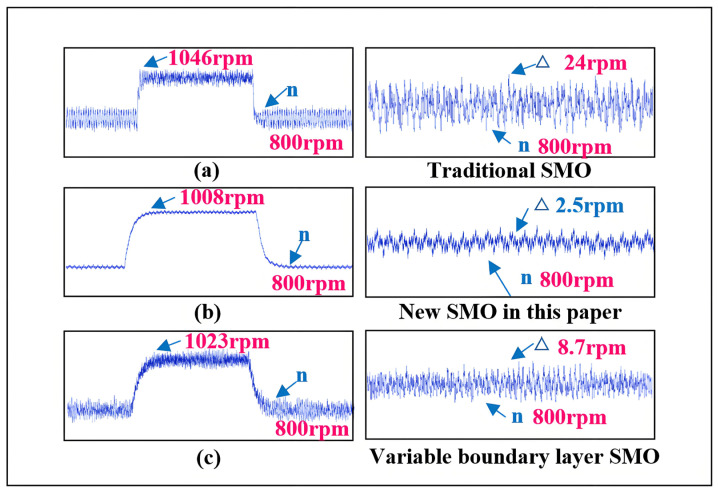
Comparison of speed with other SMOs. (**a**) is the speed and speed error of the traditional SMO. (**b**) is the speed and speed error of the new SMO proposed in this paper. (**c**) The rotational speed and rotational speed error of the variable boundary layer SMO.

Comparing the traditional SMO with the variable boundary layer-based SMO, the proposed method demonstrates smaller overshoot and reduced steady-state fluctuations in speed and position, simplifying the observer and reducing the debugging workload, as shown in Figure 18.

The traditional SMO uses the sign function as its switching function, with the selection of the boundary layer corresponding to a limiting case where *Z* = 0, resulting in an infinite slope for the switching function. In comparison to the method proposed in this paper, the traditional SMO exhibits a more pronounced overshoot in speed and steady-state fluctuations in both speed and position. Moreover, the introduction of an LPF and the compensation for its phase lag complicate the design and implementation of the traditional observer, thereby increasing the debugging workload.

In contrast, the variable boundary layer SMO employs the sine function as its switching function. This approach reduces system complexity and enhances convergence speed without introducing an LPF. However, a significant amount of computation is still required to select and define the boundary layer in order to determine the optimal boundary layer parameters. Therefore, although the variable boundary layer SMO has advantages in certain aspects, it still necessitates extensive calculations for the optimization of the boundary layer selection.

### 5.2. Verification of Self-Adjusting Error Factor SMO Under Sudden Load Increase

#### 5.2.1. Feasibility Verification During Sudden Load Increase

**Figure 19:**
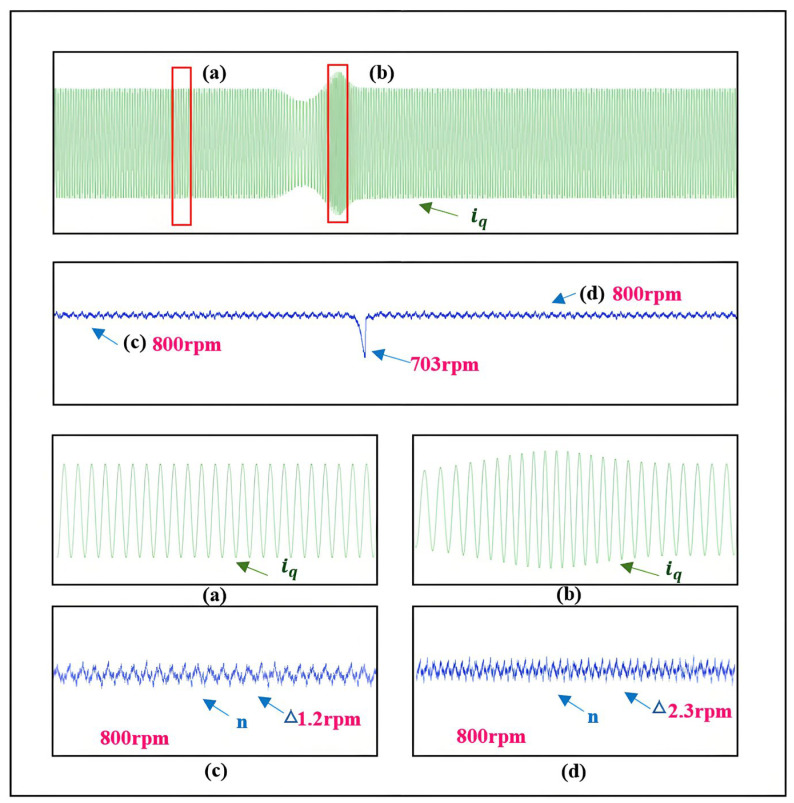
Experimental results during sudden load increase. (**a**) is the change of *q*-axis current in normal conditions. (**b**) is the change of *q*-axis current during the sudden increase of load. (**c**) is the speed change in normal circumstances. (**d**) is the speed change in the process of sudden load increase.

Analogous to the simulation and the applicable speed range of the SMO, the motor’s initial speed is set to 800 r/min, with a sudden load increase of approximately 5 Nm at 3 s. As shown in Figure 19, during the sudden load increase, the speed drops by about 97 r/min. Although the magnitude of the speed change is significant, it quickly converges back to the target speed, with steady-state fluctuations around 1.2 r/min. The estimated speed status during the sudden load increase validates the feasibility of the proposed SMO with automatic boundary layer adjustment under sudden load conditions.

#### 5.2.2. Effectiveness Verification of Self-Adjusting *Z* Value During Sudden Load Increase

The effectiveness verification here is similar to that in the previous section, comparing observation effects with the same experimental setup as in the speed increase effectiveness verification.

**Figure 20:**
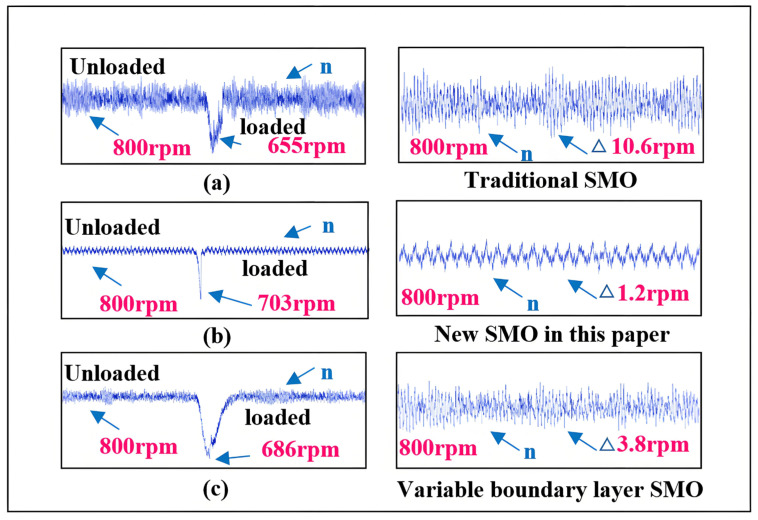
Comparison of speed for different *Z* values during sudden load increase. (**a**) the speed and speed error of the traditional SMO sudden increase load, (**b**) the speed and speed error of the new SMO sudden increase load proposed in this paper. (**c**) Speed and speed error of variable boundary layer SMO sudden increase load.

As shown in Figure 20, the curves represent the estimated speed during sudden load increase and the steady-state curves for estimated speed and estimated position and position error. According to the estimated speed curve, compared to the self-adjusting *Z* value case, when *Z* is set to 0.5, the response speed is slightly faster, but the decrease in speed is more pronounced, leading to larger steady-state fluctuations. When *Z* is set to 2, the excessive *Z* value significantly compromises the motor’s load-bearing capacity and dynamic performance, resulting in a notably longer convergence time back to the target speed after the sudden load increase. Based on the estimated position curve after the sudden load increase, the self-adjusting *Z* value sceneexhibits faster response speed and smaller steady-state fluctuations compared to the fixed *Z* value scene.

#### 5.2.3. Effectiveness Verification of the Novel SMO During Sudden Load Increase

**Figure 21:**
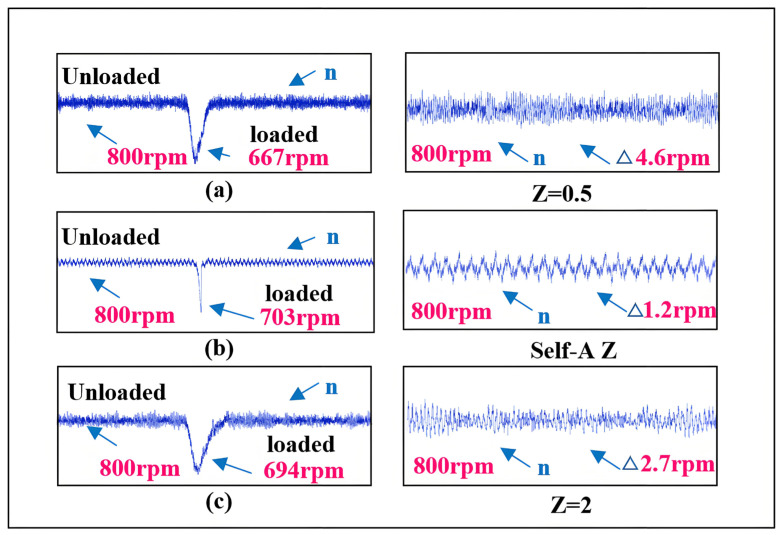
Effectiveness verification of the proposed SMO during sudden load increase. (**a**) is the speed and speed error of the sudden increase load when *Z* = 0.5, (**b**) is the speed and speed error of the new SMO sudden increase load proposed in this paper. (**c**) is the speed and speed error of the sudden increase load when *Z* = 2.

Comparing the observation effects of the self-adjusting *Z* value SMO with other SMOs, the experimental setup remains consistent with that used in the speed increase effectiveness verification. As shown in Figure 21, the curves represent the estimated speed during sudden load increase and the steady-state estimated speed curve after the load increase.

In summary, the optimal range for the *Z* value is between 0.5 and 2. Combining the error function curves corresponding to different *Z* values in Figure 2 with the aforementioned experiments, it can be analyzed that when the *Z* value is small (e.g., *Z* = 0.5), the transition region is very narrow, indicating that the system state rapidly reaches the sliding mode state as it approaches the sliding surface, resulting in quick switching on the sliding surface. While this is suitable for high dynamic control, it can lead to chattering. When the *Z* value is relatively large (e.g., *Z* = 2), the transition region is wider, meaning that the system state enters a smooth transition region as it approaches the sliding surface, allowing for slower switching changes that effectively reduce chattering, though the dynamic response may slow down, causing waveform distortion. Therefore, an optimal *Z* value exists between these two extremes (e.g., *Z* = 1.5), where adaptive changes in the error factor can better satisfy the requirements for improving system stability and dynamic response.

### 5.3. Comparative Experimental Analysis

#### 5.3.1. Quantitative Influence Experiment of Parameter Perturbation on System Efficiency

In order to better clarify how parameter modification affects efficiency, such as the change in stator resistance caused by temperature fluctuation and the change in inductance and flux linkage with time, experiments are designed to compare the traditional super-twisted SMO (fixed gain η), FNN-STASMO and the neural network self-tuning SMO proposed in this paper.

By setting the scene simulation parameters as follows, in the high temperature simulation environment of scene 1, the stator resistance Rs is artificially increased by 50% to simulate the 80 °C condition. In the demagnetization simulation of scenario 2, the flux linkage of the permanent magnet is reduced by 20%; in the inductance saturation environment of Scenario 3, the quadrature-axis inductance is reduced by 30%. The test indexes are system comprehensive efficiency ($\eta_{sys}=\frac{P_{out}}{P_{in}}$), total harmonic distortion (THD), and inverter switching loss. The experimental results are shown in Table 2.

The proposed method can still maintain high efficiency under parameter perturbation, with an average increase of 4.5~5.0%. This is mainly attributed to the fact that:

(1) The dynamic gain regulation of the neural network suppresses the current harmonics and reduces the THD by 40–50%.
(2) The adaptive observer reduces the switching loss of the inverter by 25~30%.

The quantitative relationship between parameters and efficiency was verified by linear regression analysis. When *p* < 0.01, the correlation coefficient between ΔRs and ηsys was −0.92, which proves that the change in resistance has a significant negative impact on efficiency, and the proposed method weakens this correlation through dynamic compensation.

#### 5.3.2. Performance Analysis

The control group was compared with the above settings, STSMO and FNN-STASMO, and the experimental group: the SMO in this paper. Test platform: PMSM physical bench, power level covering 1 kW/5 kW/10 kW. The test scenario type is steady-state operation: $R_{s}$ fluctuation ± 30%, $L_{q}$ ± 20%, the load is abruptly changed, and the torque step is 200% in 0.5 s. The fault injection mode is 200% torque step in 0.5 s. Table 3 summarizes the comparison of core indicators under different detection methods.

## 6. Conclusions

The objective of this research is to deeply analyze the role of the error factor *Z* in the SMO to establish selection criteria for *Z* and to construct a neural-network-error-factor model capable of automatic adjustment based on motor operating conditions. This paper systematically explores the impact of *Z* on current, speed, and position estimation in the SMO. Neural network algorithms and extensive experimental data validate the patterns, thereby establishing the selection criteria for *Z*. Based on this, a novel method is proposed to dynamically determine the error factor by estimating the rates of change in current, speed, and position errors, achieving automatic adjustment of the error factor. This ensures accurate position and speed observation of the PMSM while effectively suppressing chattering phenomena in the SMO, significantly reducing the workload required to select the optimal *Z* value. Simulation and experimental results demonstrate that this method can reliably adaptively adjust the error factor under varying conditions, balancing the trade-off between chattering suppression and control accuracy. Compared to other boundary layer determination methods, the design process workload is significantly reduced, making the selection of *Z* values more straightforward and providing high practical engineering application value.

## Figures and Tables

**Table 1:** PMSM Parameters.

Parameters	Parameter Value
pole-pairs number	4
stator winding resistance (Ω)	2.8750/per phase
magnetic flux (Wb)	0.00175
*d*-axis inductance (mH)	0.0085
*q*-axis inductance (mH)	0.0085
$\mathrm{rotational}\;\mathrm{inertia}\;(kg\cdot m^{2}$)	0.001
friction coefficient	0
remanent magnetism (20 °C) (T)	1.2

**Table 2:** Comparison of system efficiency with parameter perturbation.

Scene	Method	$\eta_{sys}$ (%)	THD (%)	*P_loss_* (W)	*t_response_* (ms)
$R_{s}+50\%$	STSMO	84.1	8.7	45.2	15.2
	FNN-STASMO	86.3	6.5	38.7	12.9
	NN-STASMO	88.9	4.3	32.1	9.8
$\psi_{f}-20\%$	STSMO	83.4	9.2	47.8	16.5
	FNN-STASMO	85.1	7.8	41.2	13.4
	NN-STASMO	87.6	5.1	35.4	10.2
$L_{q}-30\%$	STSMO	81.9	10.5	53.6	18.7
	FNN-STASMO	84.2	8.1	45.3	14.1
	NN-STASMO	86.2	6.8	39.7	11.5

**Table 3:** Comparison of core indicators under different detection methods.

Evaluating Indicator	ILC-STASMO	FNN-STASMO	This Article SMO	Test Method
position error RMSE	0.023 ± 0.005 rad	0.017 ± 0.003 rad	0.009 ± 0.002 rad	Tukey HSD
maximum overshoot	12.7%	8.9%	4.2%	Welch ANOVA
Calculation delay	85 μs	120 μs	92 μs	Kruskal-Wallis
Parameter sensitivity index	0.78	0.65	0.32	covariance analysis

## Data Availability

Data is contained within the article.

## References

[1] Guo B., Su M., Wang H., Tang Z., Liao Y., Zhang L., Shi S. (2020). Observer-based second-order sliding mode control for grid-connected VSI with LCL-type filter under weak grid. Electr. Power Syst. Res..

[2] Vural B., Dusmez S., Uzunoglu M., Ugur E., Akin B. (2014). Fuel consumption comparison of different battery/ultracapacitor hybridization topologies for fuel-cell vehicles on a test bench. IEEE J. Emerg. Sel. Top. Power Electron..

[3] Li L., Liao S., Zou B., Liu J. (2024). Mechanism-Based Fault Diagnosis Deep Learning Method for Permanent Magnet Synchronous Motor. Sensors.

[4] Yang X., Yu J., Wang Q.G., Zhao L., Yu H., Lin C. (2019). Adaptive fuzzy finite-time command filtered tracking control for permanent magnet synchronous motors. Neurocomputing.

[5] Zhang X., Zhang L., Zhang Y. (2018). Model predictive current control for PMSM drives with parameter robustness improvement. IEEE Trans. Power Electron..

[6] Xu B., Shi G., Ji W., Liu F., Ding S., Zhu H. (2017). Design of an adaptive nonsingular terminal sliding model control method for a bearingless permanent magnet synchronous motor. Trans. Inst. Meas. Control.

[7] Orlowska-Kowalska T., Wolkiewicz M., Pietrzak P., Skowron M., Ewert P., Tarchala G., Krzysztofiak M., Kowalski C.T. (2022). Fault diagnosis and fault-tolerant control of PMSM drives–state of the art and future challenges. IEEE Access.

[8] Ye S., Yao X. (2020). An enhanced SMO-based permanent-magnet synchronous machine sensorless drive scheme with current measurement error compensation. IEEE J. Emerg. Sel. Top. Power Electron..

[9] Wekhande S., Agarwal V. (2006). High-resolution absolute position Vernier shaft encoder suitable for high-performance PMSM servo drives. IEEE Trans. Instrum. Meas..

[10] Xu B., Zhang L., Ji W. (2021). Improved non-singular fast terminal sliding mode control with disturbance observer for PMSM drives. IEEE Trans. Transp. Electrif..

[11] Mohan H., Pathak M.K., Dwivedi S.K. (2020). Sensorless control of electric drives—A technological review. IETE Tech. Rev..

[12] Adeli M., Hajatipour M., Yazdanpanah M.J., Hashemi-Dezaki H., Shafieirad M. (2022). Optimized cyber-attack detection method of power systems using sliding mode observer. Electr. Power Syst. Res..

[13] Wang Y., Xu Y., Zou J. (2019). Sliding-mode sensorless control of PMSM with inverter nonlinearity compensation. IEEE Trans. Power Electron..

[14] Liang D., Li J., Qu R., Kong W. (2017). Adaptive second-order sliding-mode observer for PMSM sensorless control considering VSI nonlinearity. IEEE Trans. Power Electron..

[15] Lin S., Zhang W. (2017). An adaptive sliding-mode observer with a tangent function-based PLL structure for position sensorless PMSM drives. Int. J. Electr. Power Energy Syst..

[16] Zhou A., Qu B.Y., Li H., Zhao S.Z., Suganthan P.N., Zhang Q. (2011). Multiobjective evolutionary algorithms: A survey of the state of the art. Swarm Evol. Comput..

[17] Zhang X., Li H., Yang S., Ma M. (2017). Improved initial rotor position estimation for PMSM drives based on HF pulsating voltage signal injection. IEEE Trans. Ind. Electron..

[18] Oliva J.D.J.R., Ojeda M.P., Llanes J.S., Valle A.T. (2023). Integrated unavailability analysis including test degradation and efficiency, components ageing and common cause failures. Ann. Nucl. Energy.

[19] Yin Z., Zhang Y., Cao X., Yuan D., Liu J. (2021). Estimated position error suppression using novel PLL for IPMSM sensorless drives based on full-order SMO. IEEE Trans. Power Electron..

[20] Zaky M.S., Metwaly M.K., Azazi H.Z., Deraz S.A. (2018). A new adaptive SMO for speed estimation of sensorless induction motor drives at zero and very low frequencies. IEEE Trans. Ind. Electron..

[21] Saadaoui O., Khlaief A., Abassi M., Tlili I., Chaari A., Boussak M. (2019). A new full-order sliding mode observer based rotor speed and stator resistance estimation for sensorless vector controlled PMSM drives. Asian J. Control..

[22] Gude S., Chu C.C. (2019). Dynamic performance enhancement of single-phase and two-phase enhanced phase-locked loops by using in-loop multiple delayed signal cancellation filters. IEEE Trans. Ind. Appl..

[23] Li X., Zhan S., Guo F., Zhuang Z., Zhang H., Liao H., Qu L. (2022). Chattering suppression of the sliding mode observer for marine electric propulsion motor based on piecewise power function. Front. Energy Res..

[24] Gong C., Hu Y., Gao J., Wang Y., Yan L. (2019). An improved delay-suppressed sliding-mode observer for sensorless vector-controlled PMSM. IEEE Trans. Ind. Electron..

[25] Du S., Liu Y., Wang Y., Li Y., Yan Z. (2023). Research on a Permanent Magnet Synchronous Motor Sensorless Anti-Disturbance Control Strategy Based on an Improved Sliding Mode Observer. Electronics.

[26] He H., Gao J., Wang Q., Wang J., Zhai H. (2024). Improved Sliding Mode Observer for the Sensorless Control of Permanent Magnet Synchronous Motor. J. Electr. Eng. Technol..

[27] Jin H., Zhao X. (2019). Approach angle-based saturation function of modified complementary sliding mode control for PMLSM. IEEE Access.

[28] Wang G., Zhang H. (2022). A second-order sliding mode observer optimized by neural network for speed and position estimation of PMSMs. J. Electr. Eng. Technol..

[29] Pu R., Qiao H., Li H., Luo S. (2022). Research on vector control strategy of three-phase PMSM based on PR controller. Proceedings of the 2022 International Conference on Mechanical and Electronics Engineering (ICMEE).

